# When the chains do not break: the role of USP10 in physiology and pathology

**DOI:** 10.1038/s41419-020-03246-7

**Published:** 2020-12-04

**Authors:** Udayan Bhattacharya, Fiifi Neizer-Ashun, Priyabrata Mukherjee, Resham Bhattacharya

**Affiliations:** 1 Department of Obstetrics and Gynecology, University of Oklahoma Health Sciences Center, Oklahoma City, OK 73104 USA; 2 Peggy and Charles Stephenson Cancer Center, University of Oklahoma Health Sciences Center, Oklahoma City, OK 73104 USA; 3 Department of Cell Biology, University of Oklahoma Health Sciences Center, Oklahoma City, OK 73104 USA; 4 Department of Pathology, University of Oklahoma Health Science Center, Oklahoma City, OK 73104 USA

**Keywords:** Cell biology, Cell signalling

## Abstract

Deubiquitination is now understood to be as important as its partner ubiquitination for the maintenance of protein half-life, activity, and localization under both normal and pathological conditions. The enzymes that remove ubiquitin from target proteins are called deubiquitinases (DUBs) and they regulate a plethora of cellular processes. DUBs are essential enzymes that maintain intracellular protein homeostasis by recycling ubiquitin. Ubiquitination is a post-translational modification where ubiquitin molecules are added to proteins thus influencing activation, localization, and complex formation. Ubiquitin also acts as a tag for protein degradation, especially by proteasomal or lysosomal degradation systems. With ~100 members, DUBs are a large enzyme family; the ubiquitin-specific peptidases (USPs) being the largest group. USP10, an important member of this family, has enormous significance in diverse cellular processes and many human diseases. In this review, we discuss recent studies that define the roles of USP10 in maintaining cellular function, its involvement in human pathologies, and the molecular mechanisms underlying its association with cancer and neurodegenerative diseases. We also discuss efforts to modulate USPs as therapy in these diseases.

## Key points

- USP10 is a deubiquitinase involved in diverse cellular processes, including ubiquitin recycling, the DNA damage response, stress granule formation, and recycling of cellular proteins.
- USP10 can act as a tumor suppressor or oncogene in a context-dependent manner.
- USP10 plays an important role in neurodegenerative and infectious diseases. Therefore, understanding the specific signaling roles of USP10 may have therapeutic potential.

## Introduction

Maintaining appropriate protein levels within cells is essential for optimal cellular function and survival^1^. This intracellular protein homeostasis involves both protein synthesis and degradation. About 90% of intracellular proteins, both normal and abnormal, are degraded by the ubiquitin–proteasome system, the most prominent protein-degradation pathway^1,2^. Ubiquitination, the covalent attachment of ubiquitin (Ub) to a target protein, is an important post-translational modification regulating the stability and functional activity of proteins. This process is tightly regulated by three groups of enzymes; ubiquitin-activating enzymes (E1s) that activate ubiquitin using ATP to form Ub adenylate, ubiquitin-conjugating enzymes (E2s) that transfer Ub to ubiquitin ligases (E3s), that tag target proteins with Ub molecules^3^. Monoubiquitination occurs when a single Ub molecule is attached to one lysine residue within the target protein, while polyubiquitination is the process of attaching a chain of Ub molecules to a specific lysine residue of the target. Usually, monoubiquitination of a protein serves as a signal for vesicle sorting, signal transduction, and receptor endocytosis, whereas polyubiquitination is mainly related to protein degradation^4–7^.

Ubiquitination can be reversed by deconjugation reactions mediated by the second group of enzymes—namely, the deubiquitinating enzymes (DUBs)^8^. DUBs cleave Ub from the C-terminus of target proteins and maintain intracellular Ub homeostasis; deubiquitination is necessary to provide a sufficient pool of free Ub molecules within the cell^9,10^. This deconjugation process and its cellular consequences have not been extensively investigated to date.

Around 100 DUBs have been identified in humans, many of which remain poorly characterized with unknown functions^11^. Similarly, despite many studies on the role of ubiquitinating enzymes in human disease, there has been limited effort to understand the role of DUBs in disease^11,12^. DUBs affect multiple cellular processes, including DNA repair, and cell cycle regulation^13,14^. DUBs also remove Ub molecules from chromatin, more specifically from H2A and H2B, and can regulate gene expression^15^. Since DUBs play a major role in DNA damage repair, mutations in genes encoding the DUBs ultimately affect cell proliferation and thus are important in tumorigenesis^16,17^. DUBs also play an important role in infectious diseases; both the host and the pathogen can exploit DUBs to modulate the immune response^12,18,19^. For example, the host controls inflammatory responses via the cylindromatosis lysine 63 deubiquitinase (CYLD), a DUB responsible for regulating the nuclear factor kappa B (NF-κB) pathway^20–24^. The intracellular pathogen *Leishmania donovani*, the cause of visceral leishmaniasis, exploits the host DUB A20, an NF-κB-responsive gene, to inhibit Toll-like-receptor 2 (TLR2)-mediated signaling in macrophages^19,25,26^. Additional reports indicate that DUBs are important in some neurodegenerative diseases^27^.

DUBs are a large group of enzymes that fall into six distinct subfamilies based on their sequence and functional similarities: (i) ubiquitin C-terminal hydrolases (UCH), (ii) ubiquitin-specific peptidases (USP), (iii) Jab1/Pab1/MPN domain-containing metalloenzymes (JAMM), (iv) Otu-domain ubiquitin aldehyde-binding proteins (OTU), (v) Ataxin-3/Josephin, and (vi) monocyte chemotactic protein-induced proteases (MCPIPs). Among these, USPs are the largest family, consisting of over 50 members in humans^10^. Herein, we focus on a specific USP, namely USP10, and its role in cellular processes and human disease. We also discuss the ongoing development of USP10 inhibitors and their potential therapeutic role.

## USP structure and function

In this section, we will discuss the general structure of USPs, including USP10 specifically, as well as the specific protein targets of USP10. Specific USP functions depend on their structural architecture; Komander et al. reported that the USP domain, also called the catalytic domain, present in all USPs is composed of three regions which can be analogized as the fingers, thumb, and palm of a hand^28^. Structural analyses show that when Ub binds to a USP, the active site undergoes rearrangements that promote the catalytic hydrolysis of Ub from the tagged protein. In addition to the USP domain, Komander et al. identified several predicted Ub-binding domains (UBDs), a Ub-associated domain (UBA domain), a Ub-interacting motif (UIM), and a zinc finger Ub-specific protease domain (ZnF-UBP domain). The catalytic center of USPs is located in the interface between the palm and thumb regions of the USP domain^10,28^. As previously mentioned, binding of Ub leads to conformational changes in this domain; in the first step, a catalytic triad is formed in a specific alignment. Next, the active-site loop is displaced from its initial position which helps bind the C-terminal end of the target protein. Finally, the unanchored Ub is removed from the target^29–31^. USP7 has been studied in detail and is the archetype of this mechanism of action^32^. In the case of USP8, the finger domain moves outward to accommodate the globular structure of Ub^33^. Although the general mode of action is known for the USPs, the crystal structures and a detailed characterization for most of the family remain unknown. The current model for the mechanism of action is dependent on the crystal structure of USPs 7, 8, and 5; these represent a relatively small sample from a large number of enzymes in the group. Many other USPs remain to be thoroughly characterized and such studies may further refine the mechanistic model and add interesting modifications to the process.

**Fig. 1:**
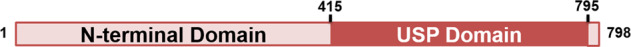
Schematic of USP10 structure. USP10 is a 798 amino acid long protein. It contains a catalytic core domain (also known as USP Domain).

Human USP10 is 798 amino acids (aa) in length and is expressed in the nucleus and cytoplasm of almost every cell. USP10 is evolutionarily conserved, and human USP10 has ~99% amino acid sequence homology with the rat and mouse proteins. The USP domain or catalytic domain (also called the core region) of USP10 is about 380-aa long and begins 415 aa from the N-terminus (Fig. 1).

Functionally, USP10 is a cysteine protease—it mediates the thiol-dependent hydrolysis of ester, thioester, amide, and/or peptide bonds formed by the carboxy-terminal glycine residue of Ub. By this hydrolysis reaction, Ub moieties are removed from the targeted proteins. The most important targets of USP10 include tumor protein p53 (TP53)^34^, cystic fibrosis transmembrane conductance regulator (CFTR)^35^, AMP-activated protein kinase alpha (AMPKα)^36^, Sirtuin 6 (SIRT6)^37^, and nuclear factor kappa B (NF-κB)-essential modulator (NEMO)^38^, all of which are involved in significant cellular processes thus explaining the central role of USP10 in cellular metabolism, signaling and tumorigenesis. USP10 also regulates autophagy by deubiquitinating Beclin1 (BECN1)^39,40^. Two proteins that are related to each other, i.e., Ras GTPase-activating protein-binding protein 1 (G3BP1) and G3BP2, bind USP10 and regulate stress granule formation in cells^41^. Other studies have shown that USP10 promotes tumor necrosis factor (TNF)-receptor-associated factor 6 (TRAF6) deubiquitination by forming a complex with TRAF family member-associated NF-κB activator (TANK) and MCPIP1 (monocyte chemotactic protein-1-induced protein 1; also known as ZC3H12A, this complex inhibits genotoxic stress- or interleukin-1-beta (IL1β)-mediated NF-κB activation^42^. The detailed functional role of USP10 and its mechanism of action, in the context of disease pathology, are discussed in the following sections.

## The USP10 homolog in yeast

USP10 is a highly conserved protein in eukaryotes, as well as being found in some lower organisms. In yeast, the USP10 homolog is UBP3, which plays a vital role in the DNA repair mechanism. The Rad4–Rad23 heterodimer is responsible for recognizing DNA damage in yeast; disruption of UBP3 results in enhanced UV resistance, increased repair of UV damage, increased Rad4 levels in Rad23 deleted cells and higher Rad4 stability. UBP3 also physically interacts with Rad4 and the proteasome, suggesting that UBP3 associates with the proteasome to assist Rad4 degradation^43^.

A second important role of UBP3 is to recycle ribosomal subunits from translational blockage. When a ribosome complex stalls during translation elongation in eukaryotes, Hel2p E3 ligase monoubiquitinates the ribosomal protein S3 (RPS3)^44^; RPS3 ubiquitination is critical for ribosome quality control^45^. RPS3 monoubiquitination is regulated by the reciprocal action between Hel2p and UBP3^44^. In mammalian cells, the reciprocal action between RNF123 E3 ligase and USP10 has an analogous role^44^.

## USP10 in cellular processes of humans

USP10 has multiple functions under normal conditions in mammalian cells. In this section, we focus on the critical cellular processes in humans that are influenced by USP10.

### USP10 as controller of the androgen receptor

The androgen receptor (AR) is a type of nuclear receptor activated by binding androgenic hormones, and it is critical for the development of male characteristics. In humans, several conditions are associated with AR, including androgen insensitivity syndrome, benign prostatic hyperplasia, and prostate cancer. Prostate development and normal prostate function are dependent on the AR signaling pathway. Thus, the AR and its modulators are considered significant factors in the progression of prostate cancer and could be potential therapeutic targets. Recent studies indicate that USP10 plays a role in modulating AR activity. USP10 deubiquitinates AR in the cytosol and enhances nuclear import and transcriptional activity of AR^46^. Overexpression of wild-type but not inactive USP10 was found to stimulate the AR activity from reporter constructs harboring selective androgen response elements, non-selective steroid response elements, or the mouse mammary tumor virus (MMTV) promoter. USP10 is therefore a cofactor that binds to the AR and stimulates the androgen response of target promoters^46^. Moreover, USP10 directly deubiquitinates H2A.Z, a variant of histone 2A (H2A) and both are necessary for AR-mediated gene expression^47^. In both hormone-sensitive and hormone-refractory prostate cancers, AR expression is observed^48–50^, and overexpression of AR is frequently observed in advanced prostate cancer^51–55^. Concurrently, USP10 overexpression is also observed in advanced prostate cancer patients and overexpression correlates with poor patient outcomes^56^. These findings identify USP10 as a critical intermediate in prostate cancer and various other clinical conditions where AR is compromised.

### USP10 in DNA damage response

**Fig. 2:**
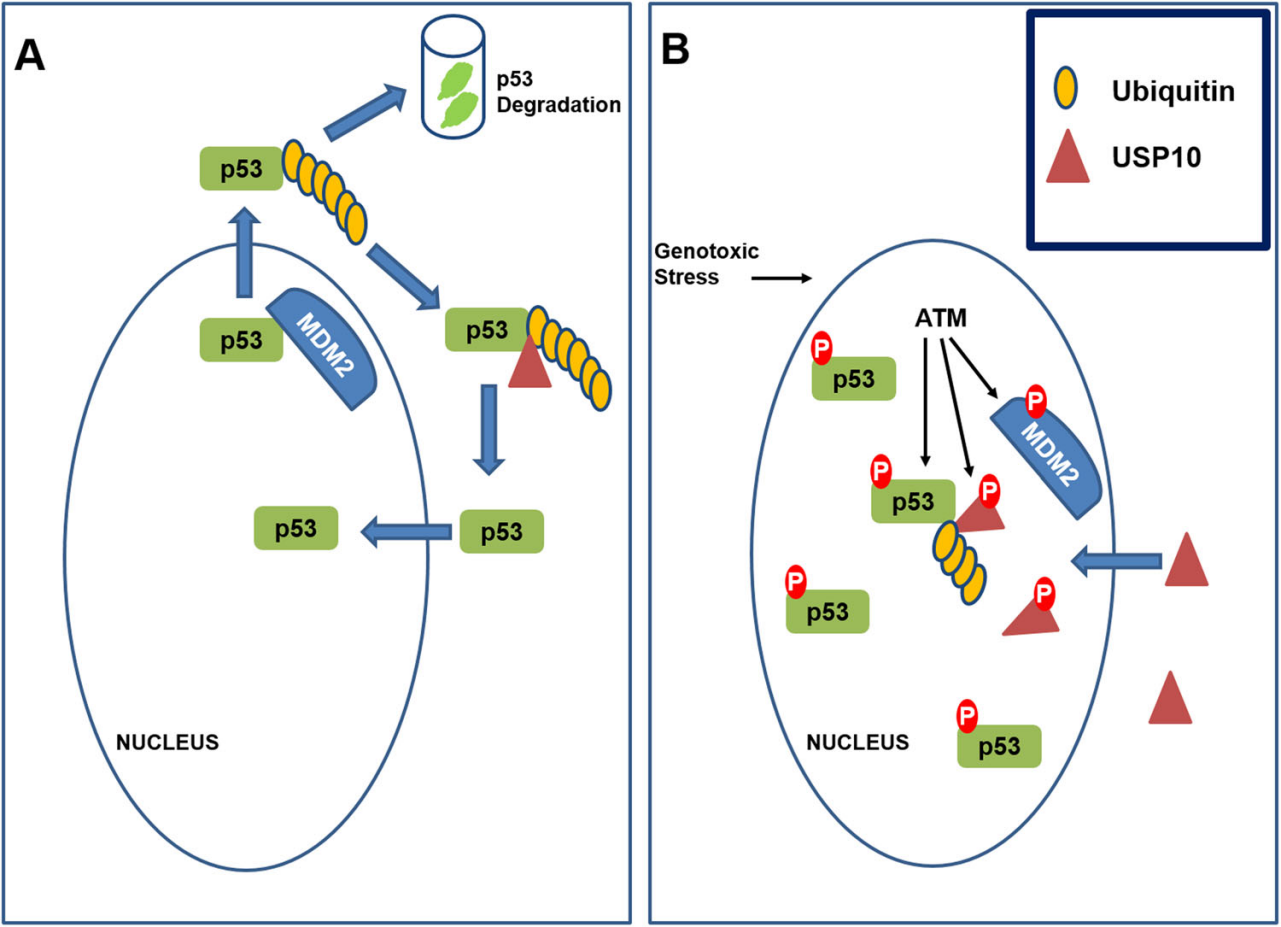
Role of USP10 in p53 stabilization. **A** Under unstressed conditions, cytoplasmic USP10 deubiquitinates p53 to prevent proteasomal degradation of p53 and allow nuclear re-entry. **B** After genotoxic stress, p53 is phosphorylated by ATM to reduce its interaction with MDM2. MDM2 gets phosphorylated by ATM making it susceptible for degradation. USP10 accumulates in the nucleus, undergoes ATM-mediated phosphorylation, and stabilizes p53 in the nucleus via deubiquitination.

The DNA damage response (DDR) protects cells against threatening mutations that arise from DNA damage. Accumulating evidence demonstrates that USPs regulate the action and stability of DDR proteins and their modulators. One well-characterized USP, specifically USP7, modulates/suppresses oxidative stress or UV-induced proliferating cell nuclear antigen (PCNA) ubiquitination thereby regulating DNA polymerase eta stability^57,58^. Additionally, USP7 is essential for regulating Rad18 stability and DNA damage tolerance^59^. USP7 regulates cellular p53 levels by deubiquitinating and stabilizing p53 or by deubiquitinating and stabilizing MDM2, a negative regulator of p53^60,61^. The role of USP10 in DDR was recently described; specifically, USP10 was identified as a regulator of p53; under unstressed conditions, USP10 deubiquitinates p53 in the cytoplasm thus countering the action of MDM2 and enabling nuclear re-entry (Fig. 2A). However, upon DNA damage, USP10 accumulates in the nucleus, is phosphorylated by ATM, and deubiquitinates p53 in the nucleus (Fig. 2B). In this context, the dual action of USP10 and USP7 may be critical for maintaining p53 stability upon DNA damage. Unlike USP7, USP10 does not target MDM2^34^ and since it stabilizes both wild-type and mutant p53, the therapeutic potential of USP10 may be relevant in specific malignancies.

### USP10 in energy sensing and autophagy

**Fig. 3:**
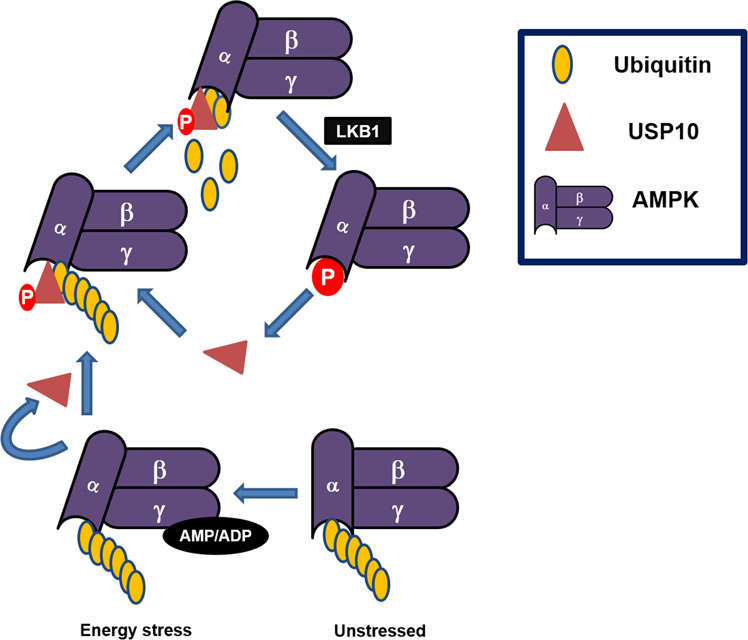
Role of USP10 in AMPK phosphorylation and activation. Under unstressed conditions, AMPKα remains ubiquitylated; ubiquitination of AMPK inhibits its phosphorylation and activation. Upon cellular stress, AMP/ADP binding to AMPKg leads to partial activation of AMPK and phosphorylation of USP10. Deubiquitination of AMPK by USP10 contributes to AMPK phosphorylation and activation by LKB1. Parallelly, activated AMPK phosphorylates USP10 thus enhancing its activity under stress thus amplifying AMPK activation.

The AMP-activated protein kinase (AMPK) is an important regulator of metabolic homeostasis. AMPK senses the cellular energy state; upon energy stress, ubiquitinated AMPK is partially activated by AMP or ADP binding and phosphorylation within the activation loop of its kinase domain. Partially activated AMPK phosphorylates USP10 at Ser76, enhancing USP10 activity to mediate AMPKα deubiquitination. The activity of USP10 facilitates phosphorylation of AMPKα at Thr172 by LKB1. This feedforward loop ensures that AMPK activation is amplified in response to energy stress^36^ (Fig. 3). The role of AMPK in promoting autophagy is well established^62,63^, and the impact of USP10 on AMPK activity and the feedforward loop suggests a potential role of USP10 in modulating autophagy. Interestingly, USP10 also regulates Beclin1^40^, a key promoter of autophagy^64^. USP10-mediated deubiquitination of Beclin1 protects against degradation thus promoting autophagy^40^. Strikingly, depletion of Beclin1 reduces both USP13 and USP10 levels^40^, suggesting the presence of a regulatory feedback loop. Although the interaction of USP10 with Beclin1 is transient, the interaction of Beclin1 with USP13 is robust therefore Beclin1 through USP13, a DUB that deubiquitinates USP10^40^ can modulate USP10 levels. Notably, there is an intricate reciprocal regulation of autophagic factors and their cognate DUBS. USP10 may therefore be integral in human pathologies associated with deregulation of autophagy. Together with established roles of AMPK activation in alleviating obesity, insulin resistance, type 2 diabetes, metabolic syndromes, neurological disorders, and cancer^63,65^, USP10 may be attractively pertinent in these pathologies.

### USP10 in NOTCH signaling

Notch signaling is important for determining the sprouting behavior of endothelial cells (EC) in vascular morphogenesis. USP10 regulates Notch signaling during angiogenic spouting by interacting with and by stabilizing the NOTCH1 intracellular domain (NICD1) in ECs^66^. Studies in mice retina, a well-established model of Notch-dependent angiogenesis, revealed that deletion of USP10 in ECs increased EC density and sprouting; recapitulating phenotypes of reduced Notch signaling. Overexpression of NICD1 resulted in retinal vascular defects that were partially restored by USP10 inhibition^66^. These findings suggest that USP10 regulates Notch-dependent vascular morphogenesis. Retinal development and vascular homeostasis is largely controlled by Notch signaling^67^, deregulation of which can lead to retinal dysplasia and neovascularization-associated diseases, such as diabetic retinopathy^67,68^ and age-related macular degeneration^67^. Therefore, eye-related pathologies like diabetic retinopathy that are characterized by upregulated Notch signaling^68^ may benefit from USP10 targeting.

### USP10 in ribosome recycling and stress granule formation

The USP10-G3BP1 complex is important for deubiquitinating ribosomal proteins (RPS2, RPS3, and RPS10) and disassembly of stalled/collided ribosomes. The action of USP10 prevents lysosomal degradation of 40S subunits and ensures recycling of ribosomal subunits^44,69^. Although USP10 may not be required for initial ribosome quality control (RQC) function^69^, it may be essential for detachment of ribosomes from mRNA, and splitting, or turnover of ribosomal subunits in the event of ribosome stalling. Ribosome recycling is essential for the viability of cells^70^, malfunctioning of recycling is associated with a mistranslation of proteins within cellular compartments and can cause unfolded protein responses, leading to autophagy—especially relevant in aging^71^. Presently, it is unclear if the USP10 function is absolutely essential or can be compensated by other DUBs that facilitate the recycling of ribosomal subunits. If essential, then targeting USP10 may show some adverse effects. However, transient or moderate downregulation may promote an unfolded protein response that can lead to cell death which may be useful in specific malignancies where it is upregulated. Stress granules are assemblies of untranslated messenger ribonucleoproteins (mRNPs). They contain stalled translation preinitiation complexes that are assembled into discrete granules by specific RNA-binding proteins i.e, G3BP. USP10 binds G3BP proteins and thus can minimize stress-related damage and promote cell survival^72^. This mechanism is important in neurodegenerative diseases and Tau pathology which is discussed in more detail below.

### USP10 in hematopoiesis

USP10 is crucial for hematopoiesis; in a study in mice, all USP10 knockout mice died within 1 year due to bone marrow failure with pancytopenia i.e., deficiency of all three cellular components—red cells, white cells, and platelets. Bone marrow failure in these mice was associated with remarkable reductions of long-term hematopoietic stem cells (LT-HSCs) in the bone marrow and liver^73^.

Thus, USP10 is involved in diverse cellular functions, and its involvement in multiple human pathologies is not surprising; USP10-associated pathologies include cancer and neurodegenerative diseases. In the following sections, we will address the role of USP10 in various disease states.

## USP10 and cancer

USP10 has crucial functions in tumorigenesis; it controls cell viability, differentiation, and apoptosis. USP10 is overexpressed in multiple malignancies including certain breast cancers and glioblastoma, while it is under-expressed in other malignancies including gastric carcinoma, and colon and lung cancers, suggesting that the role of USP10 in cancer is context-dependent. Below we elaborate the context-dependent roles of USP10 in cancer.

### USP10 as tumor suppressor

**Fig. 4:**
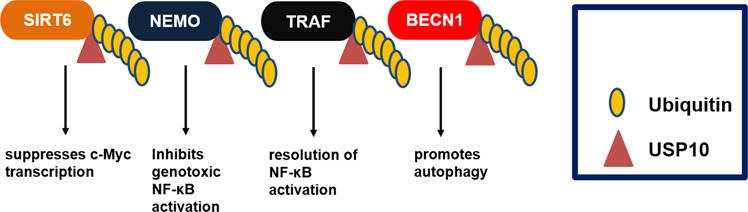
Role of USP10 in regulating various cellular functions. USP10 interacts with and deubiquitinates various proteins, including SIRT6, NEMO, TRAF, and BECN1. The activity of USP10 towards these targets mediates various cellular functions.

To understand the role of USP10 in colon cancer Lin et al. took a proteomic approach and identified USP10 as a SIRT6-interacting protein. USP10 suppressed SIRT6 ubiquitination thus protecting it from proteasomal degradation^37^. Mechanistically, USP10 antagonized the transcriptional activation of the c-Myc oncogene through SIRT6, as well as TP53, to inhibit cell cycle progression, cancer cell growth, and tumor formation (Fig. 4). Additionally, human colon cancer tissues had reduced levels of both USP10 and SIRT6 compared to adjacent normal tissue^37^, suggesting a role of USP10 as a tumor suppressor.

Zeng et al. assessed USP10 expression levels in gastric carcinoma (GC). USP10 expression was lower in GC cells than in immortalized gastric epithelial cells. In a patient population USP10 expression negatively correlated with gastric wall invasion (*P* = 0.009), nodal metastasis (*P* = 0.002), and TNM stage (*P* = 0.0001), and expression of USP10 was lower in GC tissues than in non-cancerous mucosae (*P* < 0.05). Finally, poor prognosis in GC patients was associated with negative USP10 expression thus suggesting USP10 may have prognostic significance in GC^74^.

Sun et al. used co-immunoprecipitation to demonstrate an interaction between USP10 and the phosphatase and tensin homolog (PTEN) protein; they concluded that USP10 inhibits lung cancer cell growth and invasion by upregulating PTEN^75^. Similarly, Zhang et al. revealed that USP10 interacts with and stabilizes MutS Homolog 2 (MSH2) in lung cancer cells. MSH2 levels positively correlated with USP10 levels in lung cancer cell lines. The USP10-MSH2 pathway regulates the DNA damage response^76^; knockdown of USP10 in lung cancer cells increased cell survival and decreased apoptosis following treatment with DNA-damaging agents. In an analysis of 148 patients with non-small cell lung cancer (NSCLC) (101 men and 47 women; age range, 40–76 years; mean age, 60.0 ± 8.2 years), there was no correlation between USP10 mRNA expression and clinicopathologic features, including age, sex, tumor size, TNM stage, and tumor cell differentiation. However, USP10 protein expression was downregulated in clinical NSCLC tissue samples compared with non-cancerous lung tissues^77^.

USP10 inhibits hepatocellular carcinoma (HCC) growth in vivo by inhibiting the mTOR signaling pathway. Additionally, USP10 is significantly downregulated and associated with poor prognosis in HCC. Mechanistically, USP10 stabilizes PTEN and AMPKα by inhibiting polyubiquitination of these proteins in HCC which leads to inhibition of AKT and mTOR activation^78^.

Han et al. showed that loss of USP10 (and p14ARF) expression is associated with tumor progression and poor prognosis in epithelial ovarian cancer; USP10 knockdown in ovarian cancer cells led to increased proliferation and clonogenicity^79^. In patients with small intestinal adenocarcinoma, loss of USP10 and p14ARF is associated with poor prognosis. In 195 surgically resected small intestinal adenocarcinoma tumors, USP10 expression was significantly decreased compared to normal tissue. Loss of USP10 was seen in 124 out of 194 (63.9%) small intestinal adenocarcinoma samples and correlated with a higher pT stage (*P* = 0.044), lymphatic invasion (*P* = 0.033), and the absence of sporadic adenoma (*P* = 0.024) and peritumoral dysplasia (*P* = 0.019), suggesting that loss of USP10 and p14ARF can be used as prognostic markers in small intestinal adenocarcinoma^80^. More recently, Kim et al. observed USP10 and p14ARF protein expression by immunohistochemistry on a tissue microarray from 280 colorectal cancer cases. USP10 expression was lost in 18.6% of samples, and this was linked to lymphovascular invasion (*P* = 0.019) and distant metastases (*P* < 0.001). Decreased expression of USP10 correlates with unfavorable prognosis in colorectal cancer^81^.

The preceding studies show that USP10 can act as a tumor suppressor via specific cellular mechanisms. However, in other cancers, the effect is very different and is discussed in the following section.

### USP10 as oncogene

In 2006, Grunda et al. first showed that increased expression of USP10, along with survivin and thymidylate synthetase, is associated with poor survival in patients with glioblastoma multiforme (GBM)^82^. They used a novel real-time quantitative low-density array approach to identify differentially expressed genes in GBM patient tissues and concluded that USP10 expression can be leveraged as a prognostic indicator in GBM.

High expression of USP10 is also significantly associated with poor prognosis in prostate cancer patients. The effect of USP10 in prostate cancer is via its modulation of the p53-G3BP2 complex and androgen receptor signaling; USP10 associates with and increases the stability of G3BP2 by reducing polyubiquitination. Increased levels of G3BP2 inhibit p53 activity^56^ leading to uncontrolled proliferation.

In breast cancer cells, USP10 has been identified as a critical regulator of the phosphoinositide 3-kinase (PI3K) pathway. USP10 was also identified in complex with the mitogen-activated protein kinase kinase 1 (MEK1) and PTEN proteins. Functionally, USP10 stabilizes ITCH, which is an E3 ligase for MEK1; this results in degradation of MEK1 and decreased PTEN plasma membrane localization. Downregulation of USP10 decreased activation of AKT, causing decreased colony formation^83^. Additionally, USP10 is overexpressed in breast cancer, and overexpression correlates with tumor progression and poor overall patient survival^38^.

In acute myeloid leukemia (AML), ~30% of patients harbor mutations in Fms-related receptor tyrosine kinase 3 (FLT3) which usually results in internal tandem duplications (FLT3-ITD)—thus allowing AML cells to proliferate^84^. Expression of mutant FLT3-ITD in AML patients correlates with poor prognosis and decreased survival. USP10 selectively deubiquitinates and stabilizes the mutant FLT3-ITD resulting in the accumulation of FLT3-ITD promoting oncogenic function^84^.

Thus, USP10 can act as both tumor suppressor and oncogene, depending on the type of cancer. The role of USP10 is not only relevant in cancer but it also has prominent roles in neurodegenerative disease, cystic fibrosis, and some infectious disease.

## USP10 in Alzheimer’s disease and other neurodegenerative diseases

Alzheimer’s disease (AD) is the most common form of dementia and is caused by Tau aggregation in neurons. The protein TIA1 initiates Tau aggregation by inducing the formation of stress granules. Stress granule formation is prevalent in the initial stages of several neurodegenerative diseases including AD and Parkinson’s disease. Piatnitskaia et al. showed that USP10 participates in Tau aggregation in neuronal cells exposed to stress. Exposing HT22 neuronal cells to stress resulted in the formation of TIA1/Tau-positive stress granules that were severely attenuated by depletion of USP10. In this study, USP10 colocalized with Tau aggregates in the “cell body” of neurons in AD brain lesions suggesting that USP10 is important for stress granule formation in Tau pathology^85^.

Accumulation of ubiquitinated proteins is cytotoxic, especially for neurons, and can lead to apoptosis and autophagy. Generally, neurons overcome the cytotoxicity of ubiquitinated proteins by “aggresome” formation. Aggresomes refer to an assemblage of aggregated or misfolded proteins that occur when the cell’s degradation system is overwhelmed. Takashaki et al. found that USP10 inhibits ubiquitinated protein-induced apoptosis by inducing aggresome formation; USP10 interacted with p62 and this interaction augmented p62-dependent ubiquitinated protein aggregation and aggresome formation, thereby inhibiting apoptosis. USP10 induced the formation of aggresomes containing α-synuclein, a pathogenic protein in Parkinson’s disease, in cultured cells. In Parkinson’s disease brains, USP10 colocalized with α-synuclein in the disease-linked aggresome-like inclusion-bodies called Lewy bodies, suggesting that USP10 limits α-synuclein-induced neurotoxicity by promoting Lewy body formation^86^. Collectively, these findings suggest that USP10 is a critical factor to control protein aggregation, aggresome formation, and cytotoxicity in neurodegenerative diseases.

## USP10 in cystic fibrosis and lung infection

**Fig. 5:**
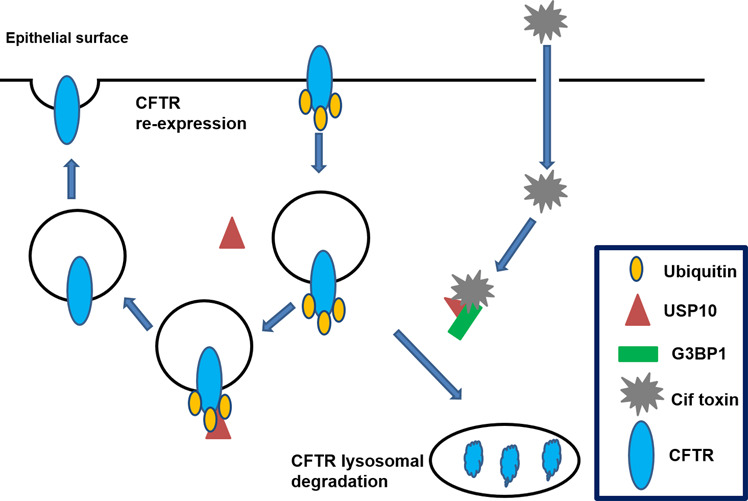
Role of USP10 on CFTR expression on the epithelial surface. Under healthy conditions, CFTR is multi-ubiquitinated and endocytosed. USP10 mediates the deubiquitination of CFTR in the endosome; preventing lysosomal degradation of CFTR and facilitating recycling to the epithelial surface. Under the pathological conditions, Cif toxin stabilizes the inhibitory effect of G3BP1 on USP10 and inhibits the recycling of the CFTR leading to reduced CFTR abundance and chloride secretion.

The cystic fibrosis transmembrane conductance regulator (CFTR) is a membrane protein that functions as a chloride channel and helps to maintain the balance of water and salt on the surface of the lung (and on other body surfaces) thereby helping in mucociliary clearance and elimination of pathogens from the lung^35^. When CFTR function is compromised, chloride is trapped in cells and the cellular surface becomes dehydrated. This results in the thick and sticky surface mucus that characterizes cystic fibrosis. The amount of CFTR on the cell surface is determined by ubiquitination-dependent CFTR endocytosis and deubiquitination-dependent recycling of CFTR to the plasma membrane of human airway epithelial cells^35^. Recently, a novel role of USP10 has been described in mediating CFTR deubiquitination in early endosomes thereby enhancing the endocytic recycling of CFTR^35^. USP10 directly interacts with and deubiquitinates CFTR resulting in increased cell surface expression of CFTR. In lung infections caused by *Pseudomonas aeruginosa*, a Gram-negative bacterium, Cif, a toxin produced by the bacterium, reduces CFTR-mediated chloride secretion by epithelial cells. Cif regulates CFTR deubiquitination in endosomes by inhibition of USP10^87^. Cif stabilizes G3BP1-mediated inhibition of USP10, which then reduces USP10-mediated deubiquitination of CFTR and increases degradation of CFTR (Fig. 5). USP10 may also be targeted by other pathogens that similarly regulate CFTR and mucociliary clearance leading to compromised immunity of the lung^35^. Expression of USP10 in human airway epithelial cells is therefore protective for CFTR abundance and chloride secretion, which is beneficial to patients with pneumonia, chronic obstructive pulmonary disease (COPD), and cystic fibrosis. Vasopressin has been shown to upregulate USP10 expression in kidney cortical collecting duct cells and increases the surface expression of epithelial Na^+^ channels through deubiquitination and stabilization of sorting nexin3 by USP10^88^. Vasopressin can also stimulate CFTR-mediated chloride secretion^89,90^, possibly via USP10 upregulation. Therefore, USP10 expression may be advantageous in cystic fibrosis patients to promote CFTR recycling to the epithelial surface. Additionally, a better understanding of the role of USP10 in the endocytic trafficking of CFTR and other ion channels has the potential to identify new targets for drug development in multiple diseases, including cystic fibrosis.

We have discussed the importance of USP10 in different cancers, in AD, Parkinson’s disease, and cystic fibrosis. In the following section, the therapeutic potential of DUB inhibitors, especially inhibitors of USP10, will be discussed in a cancer-specific context.

## Therapeutic potential of DUB inhibitors

DUBs are now considered excellent targets for drug development^91^. Many viral and bacterial DUBs have been identified in the last two decades and shown to be involved in the initiation and progression of the infection. Modulation of host DUBs by pathogenic agents is also well-characterized^88^. Based on the rapid developments in this field, the design of DUB inhibitors to treat infections is ongoing^19^.

Abnormal function and/or regulation of the ubiquitin–proteasome system (UPS) is associated with multiple malignancies. The FDA has already approved the proteasome inhibitor bortezomib (Velcade^®^) for the treatment of multiple myeloma and mantle cell lymphoma^92,93^. DUBs are an important class of molecules that regulate the UPS, as such DUB inhibitors are currently being assessed as anticancer drugs^94,95^. In the last few decades, many small molecules that specifically target DUBs have been developed and clinically tested. USPs are a highly specialized and important class of DUBs with emerging therapeutic potential, especially in cancer. Although the USP family as a whole remains largely unexplored, select USPs and their inhibitors have been the focus of recent research. For example, P5091 is a USP7-specific inhibitor that induces apoptosis in multiple myeloma cells resistant to conventional and bortezomib therapies through stabilization of TP53^96^. Pimozide and ML323, both USP1/UAF1 (USP1-associated factor 1) inhibitors, have been tested in NSCLC and osteosarcoma cells^97,98^. Pimozide sensitizes cisplatin-resistant NSCLC cells and enhances cytotoxicity of cisplatin^97^. Pimozide also targets STAT5^99^, thus, its therapeutic effect may be independent of USP targeting or, its ability to dually target STAT5 and USP1/UAF1 may be central to its potency. Further studies are required to elucidate distinct mechanisms. Another molecule EOAI3402143, a USP5/USP9x inhibitor was recently shown to induce p53-dependent FAS expression, reverse vemurafenib resistance, and decrease melanoma growth^100^. The small molecule WP1130 selectively inhibits a variety of DUBs and upregulates proapoptotic protein levels^101^. Although it was initially identified in Janus-activated kinase (JAK)–signal transducer and activator of transcription (STAT) pathway inhibitors cell-based screens, WP1130 does not inhibit JAK2 kinase activity but rather inhibits the activity of DUB enzyme^101^. It is still unclear how WP1130 impacts DUB activity and all of the DUBS affected by WP1140 have not been identified.

Spautin-1 is a promising inhibitor of USP10, and it inhibits autophagy by targeting Beclin1^40^. Inhibition of autophagy by spautin-1 enhances imatinib mesylate (IM) induced apoptosis in chronic myeloid leukemia^102^; IM is a targeted competitive inhibitor of the BCR-ABL tyrosine kinase. The proapoptotic activity of spautin-1 is also associated with the activation of GSK-3β^102^. In canine appendicular osteosarcoma cells, spautin-1, either alone or combined with doxorubicin, decreases cell survival and colony formation^103^. Spautin-1-treated ovarian cancer cells also show decreased survival^104^. In prostate cancer, spautin-1 can inhibit EGFR signaling and induce cell death, although this effect is independent of USP10^105^. A recent study showed the potential of spautin-1 in suppressing melanoma growth by inducing reactive oxygen species-mediated DNA damage^106^. Another study by Weisberg et al. showed that two other molecules, P22077 and HBX19818 also inhibit the deubiquitinase activity of USP10 thereby promoting degradation of FLT3-ITD in cancer cells^84^. Together, these reports indicate that USP10 inhibition has significant potential in cancer therapy.

## Conclusion

It is well established that the addition and removal of Ub from target proteins are essential for normal cellular functions. Rapid advances in the field demonstrate the importance of DUBs in this process. Although the biology of USP10 is not fully characterized, our current knowledge of how USP10 maintains cellular function is expanding. USP10 is an important DUB, it is primarily a cysteine protease, with complex behavior and regulates multiple aspects of cellular function in both normal and pathological conditions. We have detailed the role of USP10 in multiple disease states and explained how USP10 modulation may be beneficial in these diseases. Current developments of small molecule inhibitors for a number of USPs provide scope for targeting USP10 for therapeutic purposes. Although we have attempted to create a complete portrait of USP10 functions in relation to human pathophysiology, basic research of the molecular mechanisms underlying USP10 functions and its distinct roles in physiological and pathological regulations will be instrumental in the characterization of USP10 as therapy.
